# Lenalidomide Desensitization in Systemic Light-Chain Amyloidosis With Multi-Organ Involvement

**DOI:** 10.14740/jocmr2303e

**Published:** 2015-08-23

**Authors:** Jack T. Seki, Naoko Sakurai, Vishal Kukreti

**Affiliations:** a Department of Pharmacy, Princess Margaret Hospital, Toronto, ON, Canada; b Leslie Dan Faculty of Pharmacy, University of Toronto, Toronto, ON, Canada; c College of Pharmacy and Health Sciences, Drake University, Des Moines, IA, USA; d Department of Medical Oncology and Hematology, Princess Margaret Cancer Centre, Toronto, ON, Canada

**Keywords:** Immunomodulation, Hypersensitivity, Rash, Heart failure

## Abstract

Limited therapeutic options are available to amyloid patients treated with many lines of therapy. Although combination therapy using lenalidomide and dexamethasone is an effective sequential regimen for systemic amyloidosis (AL), dexamethasone is often poorly tolerated in patients with cardiac involvement. Lenalidomide as single agent has modest activity, but when used in combination with dexamethasone, careful titration is needed. Dermatological adverse reactions can be problematic to patients on lenalidomide-based therapy. Lowering lenalidomide doses have not been able to consistently prevent recurrent skin toxicity. We report a patient who was neither eligible for stem cell transplant nor able to tolerate previous lines of therapy. Therapeutic dilemma arose from lenalidomide-related moderately severe skin toxicity. We enrolled the patient in the lenalidomide rapid desensitization program (RDP) with success in the presence of poor cardiac reserve and renal impairment. No recurrence of skin rash was observed during the course of therapy. To the best of our knowledge, this was the first AL patients who received and tolerated RDP well, despite multi-organ impairments. The target dose may be achieved based on individual patient’s ability to tolerate RDP. Incremental dose increase can be applied in future dates without risk of rash recurrence.

## Introduction

Multi-organ involvement is a common clinical presentation in patients with light-chain systemic amyloidosis (AL) [1]. About 70% of systemic AL present with rapidly progressive cardiac involvement resulting in dysrhythmias, heart failure and high mortality rates [2]. The primary treatment goal for this disorder is to therapeutically target the clonal plasma cell and obtain complete hematological responses and subsequently organ response with improvement in mortality [3]. Immunomodulating agent such as lenalidomide often given in combination with steroid, is a reasonable treatment approach in patients who have received previous lines of treatments such as melphalan-containing chemotherapy, autologous stem cell transplantation, and bortezomib-containing regimens [4-9]. In a largest cohort of monotherapy lenalidomide-treated AL patients, 10 (43%) developed dermatological adverse reactions [10]. The skin rashes were described as morbilliform and urticarial in patterns of minor or moderate in severity. We described herein a young female patient who developed moderately severe maculopapular skin rash from lenalidomide single agent for cardiac AL, and was successfully re-challenged using a rapid desensitization program (RDP) protocol previously developed for a multiple myeloma patient [11].

## Case Report

This 46-year-old patient was initially presented with a 2 - 3 months history of heart failure symptoms (NYHA class 2), and was subsequently diagnosed with kappa light-chain amyloid using an endomyocardial biopsy in May 2009. She developed 1 - 2 degree of AV block, two episodes of syncope and non-sustained ventricular tachycardia, which warranted a dual chamber DDR type defibrillator implant in June 2009.

**Figure 1:**
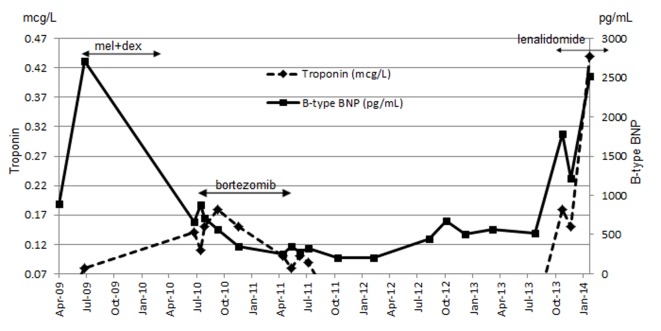
Combined results of troponin and BNP throughout the course of treatment.

**Figure 2:**
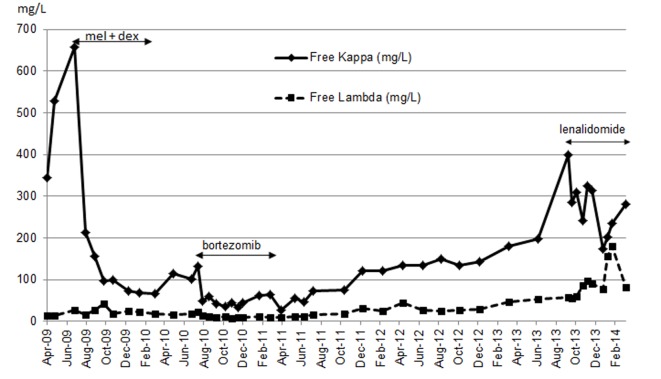
Free kappa and lambda during the course of chemotherapy.

Our patient was not eligible for autologous stem cell transplant, but instead she received melphalan and dexamethasone which began in July 2009. She developed thrombocytopenia on the seventh cycle as dose limiting toxicity that required therapy discontinuation. She had achieved partial response (PR) as per hematologic response criteria (Table 1) [4]. Although BNP levels (baseline 2,708.1 pg/mL) continued to decline from the start of treatment, troponin level was persistently elevated (baseline 0.08 μg/L) (Fig. 1). Her disease progressed by July 2010. Proteasome inhibitor (PI) bortezomib and dexamethasone weekly combination regimen was initiated. Granted that this chemotherapy regimen has made very good partial response (VGPR) by April 2011 (Table 1), she had been experiencing several side effects (grade 3) including diarrhea, severe fatigue and peripheral neuropathy. Bortezomib-related neuropathic changes cannot be ruled out, although her baseline neurological exams were unremarkable prior to the start of therapy. During this time, BNP levels were persistently decreasing, while troponin levels reached its maximum (0.18 μg/L) 2 months after bortezomib treatment began (Fig. 1). She was not tolerating bortezomib despite dose reduction to 1 mg/m^2^. PI was discontinued after completing the fifth cycle in April 2011. Due to bortezomib-related complications, she was not willing to proceed further with treatment. Interestingly during the next 29 months treatment-free period, BNP and troponin levels maintained below 672.6 pg/mL and 0.07 μg/L respectively (Fig. 1). NYHA classification was sustained at level 2. It was not until a sharp rise in her free kappa and lambda levels by October 2013 (Fig. 2), where she then consented to commence single agent lenalidomide starting at 5 mg every other day (21 days out of a 28 day cycle), in the presence of cardiac symptoms and reduced creatinine clearance of less than 30 mL/min. On the fifth day of therapy at home, she developed a moderately severe rash (grade 3 - 4) which was maculopapular in nature affecting more than 50% of body surface area involving her mouth, palms, soles, underneath the axillae, groin, back of her legs and also on the torso. She experienced pruritus, fatigue and mild weight loss. The rash was completely resolved within 3 days after discontinuation of lenalidomide. An in-patient oral RDP was used due to limited therapeutic options for this patient and the desire to maximize lenalidomide therapy in December 2013 (Supplementary 1, http://www.jocmr.org; Table 2). She received a cumulative dose of 2.65 mg of lenalidomide. The entire procedure took 4.5 h to complete in the in-patient setting. She was stable and did not experience any adverse events. She began with a dose of 1 mg every other day over the next 8 days. She also tolerated dose escalation well with lenalidomide 2 mg every other day, and over the next 21 days. As time progressed, her dose was increased to lenalidomide 5 mg every other day in January 2014 without any complications. She had achieved a PR (Table 1).

**Table 1:** Hematological Responses of Our Patient at Various Stages of Treatments Based on Girnius S. Seldin DC. JCO. 2013

Chemotherapy	Date	Kappa	Lamda	K/L	dFLC	Hematological response
Mel + dex July 7, 2009 - March 16, 2010	July 7, 2009	657	27.3	24.07	629.7	PR
	March 16, 2010	65.6	17.1	3.84	48.5	
Bortezomib July 27, 2010 - May 24, 2011	July 27, 2010	133	23.5	5.66	109.5	VGPR
	April 12, 2011	27.3	9.6	2.84	17.7	
Lenalidomide October 15, 2013 - April 19, 2014	October 15, 2013	309.9	59.3	5.23	250.6	PR
	January 7, 2014	173.1	77.6	2.23	95.5	

**Table 2:** Incremental Increase in Lenalidomide Dosing for Desensitization and Patient Vitals Monitoring Table

Dose#	Stock solution concentrations	Dose (mg)	Amount given (mL) by mouth	Time the dose given	BP (mm Hg)	HR per min	RR per min	Temp (°C)	O_2_ Sat (%)
Baseline				14:00	104/66	69	16	36.6	97
1	Solution D (0.001 mg/mL)	0.00025 mg	0.25	14:15	105/66	70	16	36.9	98
2		0.00125 mg	1.25	14:30	103/64	71	16	36.5	98
3		0.0025 mg	2.5	14:45	104/65	71	16	36.8	98
4	Solution C (0.01 mg/mL)	0.0125 mg	1.25	15:00	102/62	70	16	36.9	98
5		0.025 mg	2.5	15:15	101/62	71	16	37.0	99
6	Solution B (0.1 mg/mL)	0.125 mg	1.25	15:32	117/74	72	16	36.7	100
7		0.25 mg	2.5	15:45	106/69	72	16	37.1	100
8		0.5 mg	5	16:00	104/67	72	16	37.0	100
9	Solution A (1 mg/mL)	0.75 mg	0.75	16:15	105/66	72	16	37.1	100
10		1 mg	1	16:30	104/65	72	16	37.1	100
Final vitals				17:00	106/67	74	16	36.5	98
Observed patients				17:45					

Two weeks later (into her 12 weeks of lenalidomide), she presented with worsening signs and symptoms of CHF and hospitalization. She was unable to continue lenalidomide therapy, and was lost to follow-up.

## Discussion

While lenalidomide-dexamethasone combination therapy accounted for significant activity [12], the latter agent is probably harmful for our patient due to her cardiac condition [2, 13]. Single agent can be effective against AL [14]. Lenalidomide hypersensitivity in AL is well documented [10, 15], and all have developed within a month of exposure. The extent of urticarial or morbilliform rash reported in these patients, as in ours, involved more than 50% of the body surface. To avoid further potential recurrence of rashes upon retreatment, a lower dose was commonly tried based on a published paper [10]. However, in this series of AL and myeloma patient rash-avoidance strategy such as dose reduction did not work consistently well for some lenalidomide-treated patients [10]. In an analysis comparing patients taking dexamethasone with lenalidomide to those taking lenalidomide alone, no significant difference in incidence of rash was reported between the groups [16]. While antihistamines and topical steroids have been suggested to manage mild localized rashes [17], no published evidence supported antihistamine as pre-medication to reduce the potential dermatological toxicity. Sviggum et al also commented that amyloid patients had the highest proportion of rashes of moderate severity. For this reason and given our previous experience, we felt compelled to initiate RDP. The process involving the preparation of the testing solution was conducted in a pharmacy biological safety cabinet. The breaking of the lenalidomide capsules and the contents were used in the reconstitution of a primary solution, from which serial dilutions were made to arrive at the desired concentrations for desensitization testing. The lowest concentration 0.001 mg/mL derived in the initial stages of production was needed to make the first testing dose 0.00025 mg dispensed in an oral syringe. The remaining nine testing doses were produced by incrementally increasing in its concentrations and therefore its respective doses (Table 2). Although the mechanism of lenalidomide-related rash remained poorly defined and not well understood by the medical community [10, 11], other studies have shown that the extracellular signal-related kinase and P13K/Akt pathways may be involved [16]. Similar mechanisms have been hypothesized linking rash to other targeted agents [18, 19]. The fact that these pathways are targeted by lenalidomide on keratinocyte growth and survival, the same might be hypothesized for the development of rash in the epidermis [16]. Type I (IgE mediated) hypersensitivity reaction has been postulated [20]. The genetic basis of adverse drug reactions has been described to explain the severe type of skin reactions such as Steven-Johnsons to share the HLA-DRB*1501, and HLA-DQB1*0602 in two patients, whereas the milder rash shares the HLADRB1*1502 and HLA DQB1*0601 genetic information in a patient [21]. While tolerance is achievable and safe, the molecular basis of occupying the receptor and preventing antibody/immune cells cross-linking, may be impart the mechanism not fully understood [22]. We also noted an interesting observation that our patient remained adverse reaction-free even after a drug-free period of 7 days has lapsed under 21 days of the 28-day lenalidomide treatment cycle, which was also reported in an earlier experience [11]. This event-free phenomenon persisted over the observed 3 months period. On the other hand, in a separate case [23], upon re-challenge a patient developed hypersensitivity reaction after a 4-month lenalidomide-free period elapsed. The duration of the drug-free period may be the key to determine the re-challenge safety threshold.

Although amyloid deposition on the skin as a disease process may contribute toward confounding skin reactions post-lenalidomide exposure, our patient did not have amyloid skin involvement.

Optimal treatment outcome in AL is distinguished by hematologic response first followed by progressive organ response and overall survival has been shown to correlate with renal response [24]. Although kidney involvement is most common in AL, heart failure is commonly attributable to patient’s death [25]. Improvement in cardiac biomarkers has been coupled with enhanced overall survival [26]. Our patient did achieve VGPR hematological response with bortezomib-related treatment which lasted until Apr 2011. However, her clinical condition deteriorated progressively with increasing in light chains in the absence of treatment over the 29 months period. Although she agreed to restart treatment with lenalidomide which began in October 2013, both NT-proBNP and troponin levels continued to spike due to aggressive evolution of disease (Fig. 1). Concurrently, her NYHA was measured in the 3 - 4 class range which was reflective of her worsening disease characterized by cardiac symptoms and poor renal function.

We believed the benefit from lenalidomide treatment that began in October 2013 for this patient was suboptimal, because she did not pursue continuation of her treatment in a timely fashion post-bortezomib termination in April 2011.

### Conclusion

Lenalidomide is an effective treatment resorted in AL patients. Although dermatological hypersensitivity reactions posed real patient challenges, remedy such as using an institutionally developed RDP circumvented these problems effectively, and safely as demonstrated in this patient and other [11]. Early intervention and uninterrupted treatments are keys to favorable clinical outcome.
